# Supplementary material for: Targeting Neutrophil Extracellular Traps for Stroke Prognosis: A Promising Path
Source: Neurol Int. 2023 Sep 28;15(4):1212–26. doi: 10.3390/neurolint15040076 (PMC10594510; doi:10.3390/neurolint15040076)
Supplement: Supplementary file 1 [file neurolint-15-00076-s001.zip › neurolint-2564051-supplementary.pdf]

Supplementary Material: Targeting Neutrophil Extracellular Traps for Stroke Prognosis: A Promising Path.

Eirini Liaptsi, Ermis Merkouris, Efthymia Polatidou, Dimitrios Tsiptsios, Aimilios Gkantzios, Christos Kokkotis, Foivos Petridis, Foteini Christidi, Stella Karatzetzou, Christos Karaoglanis, Anna-Maria Tsagkalidi, Nikolaos Chouliaras, Konstantinos Tsamakis, Maria Protopapa, Dimitrios Pantazis-Pergaminelis, Panagiotis Skendros, Nikolaos Aggelousis and Konstantinos Vadikolias

Table S1. Basic characteristics of the 33 included studies.

Author, Year of Publication	Type of study-Duration	Type of stroke	Biomarker	Patient Group & Intervention	Scale of stroke severity	Sample	Time of sampling	Main Findings	Study Weakness
Pir et al. 2022 [27]	Prospective<br><br>July 2019 – January 2021	AIS	NET-markers (H3Cit, NE, MPO) in AIS patients derived thrombi	LVO patients who underwent thrombectomy<br>*n* = 21<br>mean age = 50.73 ± 11.04<br><br><u>Group 1:</u><br>Thrombolysis followed by thrombectomy,<br>*n* = 10<br><br><u>Group 2:</u><br>Thrombectomy without thrombolysis, *n* = 11	NIHSS<br><br>TOAST classification	Arterial thrombus	During endovascular thrombectomy	Abundant NET formation in AIS patients thrombi	Small sample size<br>No control group

Cha et al.2022 [33]	Prospective<br><br>December 2017 – May 2020	AIS	Neutrophil fractions in thrombi, NE as a NETs marker	LVO patients who underwent thrombectomy<br>*n* = 75<br><u>Group 1:</u><br>Lower neutrophil fraction<br>*n* = 38<br>mean age =76.5 (61.0–84.0)<br><br><u>Group 2:</u><br>Higher neutrophil fraction<br>*n* = 37<br>mean age =78.0 (71.0–85.0)	mRS at 3 months<br><br>NIHSS at admission<br><br>TICI	Arterial thrombus	During intra-arterial mechanical thrombectomy	1. **Higher** neutrophil fraction in patients with **poor outcomes** (30.0 (IQR 20.9–33.3)) than in patients with good outcomes (23.6 (IQR 10.9–30.1)) (*p* = 0.023) at 3 months<br><br>2. NIHSS in **Lower** neutrophil Group = **13.0** [10.0–18.0] VS NIHSS in **Higher** neutrophil Group = **15.0** [10.0–20.0]<br><br>3. Successful recanalization (TICI 2b or 3**)** ***p* = 0.646** and onset-to-recanalization time **(*p* = 0.491)** did not differ between the groups<br><br>*Atrial fibrillation was more frequent in the higher neutrophil group (32/37, 86.5%) than in the lower neutrophil group (19/38, 50%)* ***(p = 0.002)***	Small sample size<br>No control group

Huang et al.2022 [30]	Observational Prospective<br><br>May 2021 – February 2022	AIS	NETs markers: MPO-DNA complex H3cit	AIS patients with NIHSS score between 3 and 20<br>*n* = 45<br><u>Group 1:</u><br>Patients receiving Edavarone and conventional treatment<br>*n* = 15<br>mean age = 62.8 ± 3.4<br><br><u>Group 2:</u><br>Conventional treatment group<br>*n* = 15<br>mean age = 62.1 ± 2.6<br><br><u>Group 3:</u><br>Control group<br>*n* = 15<br>mean age =55.2 ± 3.2	NIHSS at admission	Peripheral blood	At admission within the first 48 h after stroke onset before treatment<br><br>3 days after treatment	Serum levels of MPO-DNA, and citH3 in the conventional treatment group and Eda.B group were higher after stroke onset **($p < 0.01$)** compared to controls<br><br>MPO-DNA, and citH3 in the Eda.B group decreased on the third day after being hospitalized **$p < 0.01$**	Small sample size<br>No clinical assessement after admission
De Vries et al. 2022 [31]	Prospective multicenter<br><br>September	IS	NETs markers: MPO-DNA complexes	Patients with TIA, amaurosis fugax, or minor stroke with mRs ≤3)<br>AND	mRS	Blood sampling (Platelet-poor plasma)	Three months after experiencing neurological symptoms due to ischemia	High levels of NETs were non significantly associated with more	Small sample size<br>Sampling distal to ischemic event

	2010 – December 2014		Histone-complexed DNA fragments (histone-DNA)	stenosis of the ipsilateral carotid artery above 30% and below 70%<br><br>*n* = 182<br>mean age =67 ± 9<br><br><u>Group 1:</u><br>Younger patients, with no history of statin or antithrombotic treatment<br>*n* = 72<br><br><u>Group 2:</u><br>Older patients under statin or antithrombotic treatment prior to index event<br>*n* = 109				vulnerable atherosclerotic plaques (***p* = 0.18)**<br><br>In the subgroup of patients naive to statins or antithrombotic medication, the aboe finding was statistically significant<br>**(*p* = 0.04)**	
Datsi et al. 2022 [22]	Prospective case-control	AIS	Neutrophils count, DNAse-I, LDG	<u>Group 1:</u><br>AIS patients undergone	NIHSS at admission<br><br>TICI	Peripheral blood<br><br>Retrieved thrombi	Peripheral blood: Prior to MT<br><br>Thrombus:	. significantly elevated levels of absolute granulocyte count	Small sample size<br><br>No information about long term

	February – May 2018			MT combined with IVT<br>*n* = 35<br>median age= 77 (70.9, 26–98)<br><br><u>Group 2:</u><br>Healthy controls age matched<br>*n* = 16			During MT	(***p* = 0.007**), DNAse-I (***p* < 0.001**)<br>In AIS patients peripheral blood compared to controls<br><br>2.increased DNase-I levels (***p* = 0.045**)<br>in sera / increased NET formation in thrombi<br>(***p* = 0.032**)<br>In AIS patients with time passed from symptom onset >4.5 hours<br><br>3. significantly lower neutrophil count I patients with NIHSS <5 at admission compared to patients	patients' outcome<br><br>No information on correlation between treatment received and NETs
Witsch et al.2022 [42]	Prospective, observational single-center<br><br>July 2018 – September 2020	SAH	MPO-DNA complex	Patients with spontaneous SAH<br>*n* = 78<br>mean age =59.0 (13.0)<br><br><u>Group 1:</u><br>Patients with Delayed Cerebral Ischemia.	Glasgow Coma Scale (GCS) scores<br><br>World Federation of Neurosurgeons Scale (WFNS)	Serum	At admission (day 0)<br>and on day 4	High levels of MPO-DNA complexes in all patients with aSAH<br><br>No difference for MPO-DNA levels at admission between those with DCI and those without DCI (***p* = 0.6**) and	Only Netosis marker examined : MPO-DNA complexes<br><br>No clinical outcome measured

				n = 29<br>mean age =59.7 (12.6)<br><br><u>Group 2:</u><br>Patients with no Delayed Cerebral Ischemia.<br>*n* = 49<br>mean age =58.6 (13.3)	Hunt & Hess scale			those with and without clinical vasospasm (***p* = 0.5**).<br><br>MPO-DNA levels between admission and day 4 reduced in patients with DCI (***p* = 0.04**,) but not in those without DCI (***p* = 0.17**)<br><br>MPO-DNA levels between admission and day 4 reduced in patients with clinical vasospasm (***p* = 0.006**), but not in those without clinical vasospasm<br>(***p* = 0.5**)	No healthy control group<br><br>No systematic adjustments to other variables potentially affecting DCI
Denorme et al. 2022 [43]	Prospective<br><br>June 2018 – June 2021	AIS	Brain tissue NE, MPO, H3cit<br><br>MPO-DNA complexes<br><br>NET degradation potential (DNase activity)	Brain tissue:3 patients who died after AIS average age = 81<br><br>Peripheral blood:<br><u>Group 1:</u> Stroke patients<br>*n* = 27<br>mean age =63 ± 15	NIHSS at admission 3 (0–17)<br><br>mRS at discharge 2 (0–5)	Brain tissue samples<br><br>Plasma samples	Brain tissue samples:<br>within 24 hours postmortem for 2 samples and 48 hours<br>for 1 sample<br><br>Plasma samples:<br>within 48 hours of hospital admission	NETs are present in brain tissue from ischemic stroke patients<br><br>Both plasma H3cit and MPO-DNA levels positively correlated with stroke outcomes (*r* = 0.45, ***p* = 0.024**, and *r* = 0.507, ***p* = 0.01**, respectively)	Small sample size<br>No assessment of stroke severity at admission

				<u>Group 2:</u> Healthy donors<br>*n* = 27<br>mean age =55 ± 15					
Molek et al 2022 [41]	Prospective<br><br>Not mentioned	IS	H3cit, MPO	Anticoagulated patients with atrial fibrillation<br><br>*n* = 243<br>median age = 69	Not mentioned	Serum samples	Not mentioned	Ischemic cerebrovascular events were observed in 20 patients (1.9%/year) who had at baseline higher H3cit, and MPO levels.<br><br>The independent predictors of ischemic stroke/TIA were H3cit (hazard ratio [HR] 9.48, 95% confidence interval [CI] 3.88–22.41, ***p* < 0.0001**) and VWF (HR 1.20, 95% CI 1.11–1.49, ***p* = 0.001**).<br><br>Major bleeding (2.0%/year) and all-cause mortality (1.9%/year) were not related to NETs markers.	No multivariant analysis
Desilles et al. 2022 [19]	Prospective monocentric<br><br>March 15, 2020 – May 30, 2021	AIS	Neutrophil levels<br><br>NET markers (MPO, DNA ctH3)	<u>Group 1:</u> Consecutive COVID-19 positive patients<br>*n* = 14	NIHSS	Thrombi	At the end of endovascular therapy-retrieved thrombi	COVID-19 patients median NIHSS= 23 (17–25)<br>Non-COVID-19 patients median NIHSS= 16 (13–21)	Neutrophil and/ or NETs markers levels not quantifiable<br>Small sample size

				median age= 59 (52–72)<br><br><u>Group 2:</u><br>Non-COVID-19 patients<br>n = 16<br>median age= 67 (57-76)				No difference in neutrophils and NETs markers levels in thrombi<br>Retrieved from non-COVID-19 and<br>COVID-19 patients.	Possible endothelial changes not reported, as a counfounding factor in AIS occurrence<br><br>No clear association reported between COVID-19 infection and AIS.
Genchi et al. 2022<br>[11]	Prospective multicenter<br><br>February 2020 –March 2021 for COVID-19 patients<br><br>July 2016 – November 2019 controls	AIS	H3cit<br>MPO+ cells	<u>Group1:</u><br>Patients with COVID-19 and concomitant LVO-AIS treated with MT<br>n = 7<br>mean age = 70.9 ± 12.4<br><br><u>Group 2:</u><br>COVID-19-negative (control) patients with<br>LVO-AIS,<br>treated by MT<br>mean age =74.7 ± 9.6<br><br><u>Group3:</u>	NIHSS<br><br>mRS	Cerebral thrombi<br><br>Blood samples	Thrombi:<br>During MT procedure<br><br>Blood samples:<br>48 h from stroke symptoms onset	The number of neutrophils (MPO+ cells) was significantly higher in the thrombi of COVID-19 patients than controls (respectively, median MPO+ cells/mm^2 = 2110,<br>[IQR1754–2580] vs. 1333,<br>[IQR1060–2082], ***p* = 0.04**)<br><br>The content of NETs was not significantly different between the two groups (in COVID-19, the median citH3+ area was 8.66% of total area,	Small sample size of COVID-19 thrombi

				LVO-AIS patients with recent pre-stroke infections (present at stroke symptom onset) and not related to SARSCoV-2				[IQR3.33–12.77] vs. 8.35%, [IQR6.55–50.7] in controls, ***p* = 0.19**)<br><br>Thrombi of COVID-19 patients had a three-fold higher tNLR than control patients (Respectively, median tNLR 30.3, [IQR25.9–44.4] vs. 10.7, [IQR8.1–19.0], ***p* ≤ 0.01**)<br><br>COVID-19 patients with thrombi deriving from patients with non-SARS-CoV2 pre-existing infections at stroke onset confirmed the increased neutrophil density and the higher tNLR in the COVID-19 group	
Zeng et al. 2021 [21]	Prospective<br><br>Not mentioned	SAH	H3cit<br>DAPI	Group 1:<br>Patients with aneurysmal SAH<br>*n* = 10<br><br>Group 2:<br>Control	Hunt and Hess classification score	Blood samples	Within 24 h after SAH induction	Plasma NET formation (H3cit) was increased in patients with aneurysmal SAH ***p* = 0.0012**<br><br>Positive correlation between	- Not mentioned

				patients *n* = 10				plasma H3cit levels and SAH severity in patients with aneurysmal SAH (*r*= 0.7742, ***p* = 0.0118**)	
Abbasi et al. 2022 [23]	Single-center, retrospective<br><br>October 2016 – November 2020	AIS	H3Cit	AIS patients who underwent MT less than 8 hours from symptom onset<br><br><u>Group 1:</u> Early (≤4 hrs.)<br>*n* = 88<br>mean age =67.18 (±14.17)<br><br><u>Group 2:</u> Delayed (>4 hrs.)<br>*n* = 49<br>mean age = 64.42 (±10.7)	TICI Score	Thrombi	During MT performed less than 8 hours from symptom onset	H3Cit thrombus fractions were significantly higher in the delayed group<br><br>Early group (16.31 ± 19.88)<br>Delayed group (26.57 ± 25.9)<br>***p*= 0.03**	Study design: single-center, retrospective.<br><br>Thrombus may not always have been removed en bloc and device manipulation during the procedure may cause clot fragmentation and potential embolization, affecting the composition of retrieved clots.
Zhang et al. 2021 [38]	Prospective<br><br>**Venous blood samples:** January 2019 – January 2020	IS	NET-DNA complexes (MPO-DNA, NE-DNA, and H3Cit-DNA)<br><br>Plasma cfDNA	<u>Group 1:</u><br>AIS patients -time from symptoms onset to intravenous thrombolysis within 4.5 hours	NIHSS (before and 24 hours after thrombolysis)<br><br>TICI score	Venous blood samples<br><br>Thrombi	**Venous blood samples:** Before and 24 hours after thrombolysis	AIS patients had higher neutrophil counts than healthy subjects<br>(Controls = 59.78 ± 4.35<br>Patients = 75.24 ± 3.51) ***p* = 0.046**	Not mentioned

	**Thrombi collection**<br>May 2019 – January 2020			*n* = 60<br>median age= 68.47 ± 11.86<br><br><u>*Group 1a:*</u><br>Reduced NIHSS score 24 hours after thrombolysis-improvement group<br>*n* = 40<br><br><u>*Group 1b:*</u><br>Unchanged or increased NIHSS score 24 hours after thrombolysis- no-improvement group<br>*n* = 20<br><br><u>Group 2:</u><br>Healthy controls<br>*n* = 30<br>male =20<br>mean age =60.58 ± 8.86<br><br>**Thrombi:**			**Thrombi:** During endovascular thrombectomy	NET markers in AIS patients were escalated when compared to those in healthy individuals<br>$p < 0.01$<br><br>After thrombolysis, a significant increase in the NET markers in the no-improvement group<br>$p < 0.01$<br><br>Circulating NET markers were positively correlated with plasma levels of procoagulant biomarkers indicators (TAT, D-dimer, and Fibrinogen) and fibrinolysis inhibitors<br>$p = 0.01$	

				AIS patients who had undergone endovascular thrombectomy<br>*n* = 30<br>male = 18 (60%)<br>mean age =63 ± 10					
Zhang et al.<br>Sep 2021<br>[24]	Prospective<br><br>October 2018 – November 2020	AIS	NETs<br><br>Cf-DNA<br><br>MPO<br><br>NE<br><br>Not mentioned	<u>Group 1:</u><br>Healthy controls<br>*n* = 12<br>mean age = 35.07 ± 0.5<br><br><u>Group 2:</u> Patients with asymptomatic carotid stenosis<br>*n* = 13<br>mean age = 35.57 ± 4.0<br><br><u>Group 3:</u> Patients with symptomatic carotid stenosis<br>*n* = 12<br>mean age = 43.63 ± 0.5	Not mentioned	Blood samples	Not mentioned	NETs markers levels in plasma were higher in patients with symptomatic carotid stenosis compared with the healthy controls and asymptomatic patients.<br><br>**$p < 0.05$**	Not mentioned

Orbán-Kálmándi et al. 2021 [9]	Prospective, observational, single-centered study<br><br>June 2017–September 2020	sICH	Clot lysis assays CLA and mCLA (mimic the effect of NETs assay condition)	<u>Group 1</u>: Patients with non-traumatic intracerebral hemorrhage stroke<br>$n = 89$<br>mean age = 68 (±11.6)<br><br>**Strafication by mortality rate**<br><br>*<u>Group 1a:</u>* Survival by day 14<br>$n = 63$<br>mean age = 67 ± 12<br><br>*<u>Group 1b:</u>*<br>Non-survival by day 14<br>$n = 26$<br>mean age = 71 ± 10<br><br>**Stratification based on 3 months outcome**<br><br>*<u>Group 1a:</u>* mRS 0–1<br>$n = 15$<br>mean age = 64 ± 12	NIHSS (on admission and day 7)<br><br>ICH score<br><br>mRS (3 months after stroke)	Venous blood sample	upon admission	Severe stroke (NIHSS>10) patients presented significantly shorter clot lysis times in the presence of DNA and histone as compared to patients with NIHSS<10.<br><br>***p* = 0.032**<br><br>Shorter clot lysis times were signifivantly associated with higehr mortality in day 14 and worse funcional outcomes<br><br>***p* = 0.027**	Small sample size

				<u>*Group 1b:*</u><br>mRS 2–5<br>*n* = 32<br>mean age = 67 ± 13<br><u>*Group 1c:*</u><br>mRS 6<br>*n* = 39<br>male = 28 (72%)<br>mean age = 71 ± 10<br><br><u>Group 2:</u> Healthy controls					
Genchi et al. 2021 [29]	Prospective<br><br>March 2016 – January 2019	AIS	citH3<br><br>MPO<br><br>MPO-DNA	LVO stroke patients, treated with mt<br>*n* = 115<br>*(80 had retrieved cerebral thrombi available)*<br>median age: 74 years	NIHSS<br><br>mRS	Thrombi<br><br>Peripheral blood	Thrombi: During mechanical thrombectomy<br><br>Peripheral blood: At admission within 24 h from stroke onset	NET content was significantly increased in cardioembolic compared to large artery atherosclerosis thrombi<br>***p* = 0.04**<br><br>NET content in the thrombus was<br>found to correlate with NET content in the plasma<br>***p* ≤ 0.001**	Not mentioned
Chen et al. 2021 [12]	Prospective November 2018 –	IS	H3Cit	AIS patients undergone MT<br>*n* = 30<br>mean age: 70	NIHSS<br>TICI score<br>mRS	Thrombi sample	During mechanical thrombectomy	NETs are present in the clots of patients with LVOS	Small sample size

	November 2019								Significant heterogeneity between patients<br><br>Selection bias wherein clot samples are only available in patients who had at least partial success in clot removal.<br><br>Selection bias for techniques wherein the clot could be preserved such as stentriever as opposed to aspiration alone.
Lin et al. 2021 [36]	Retrospective cohort analyses<br><br>April 2006 – December 2019	AIS	Histone/DNA complex<br><br>Citrullinated histone H3<br><br>Cell-free DNA	iTTP patients who had ischemic strokes $n = 21$ mean age: 49.9 ± 10.9	NIHSS	Blood samples	Unknown	Comparison between patients with large ischemic strokes ($n = 10$) and small ischemic strokes ($n = 11$) revealed that patients with small stroke were older (***p* = 0.043**) and had higher plasma levels of citrullinated	Exact timing variable<br><br>No detailed analysis of potential confounders, including TOAST, stroke subtype criteria, and location of the small AIS.

								histone 3 (***p* = 0.006**) and histone/DNA complex (***p* = 0.014**) than those with large strokes.	Limited sample size.
Deng et al. 2020 [26]	Prospective<br><br>Not mentioned	AIS	H3cit<br><br>NE	Ischemic stroke patients $n = 46$<br><br><u>Group 1:</u><br>Diabete mellitus (DM)<br>$n = 9$<br><br><u>Group 2:</u><br>hyperglycemia (HG)<br>$n = 9$<br><br><u>Group 3:</u><br>normoglycemic (NG)<br>$n = 28$	Not mentioned	Thrombi	During mechanical thrombectomy	NETs were observed in thrombi retrieved from ischemic stroke patients undergoing endovascular treatment.<br><br>Neutrophil elastase stained for the confirmation of neutrophils in the thrombus is shown.<br><br>Citrullinated histone H3 content was relatively low in the normoglycemic stroke patients, while it was more variable and much higher in diabetic and acute hyperglycemic patients.	Not mentioned
Essig et al. 2020	Prospective	AIS	NE	AIS of the middle cerebral artery	NIHSS score	Thrombemboli	During MT	The number of neutrophils	Not mentioned

[32]	Not mentioned		H3Cit<br><br>MPO<br><br>DNA	(MCA), internal carotid artery (ICA) or basilar artery patients undergone successful MT<br>n = 37<br>mean age =63 (56–77)	(Median on admission 17 (13–22) and on discharge 8 (4–10))<br><br>TOAST<br><br>ASPECTS			was associated with stroke severity as assessed by the NIHSS upon admission (***p* = 0.09**) and discharge (***p* = 0.07)**<br>Neutrophil numbers were higher in thrombemboli of presumed cardioembolic origin and in thrombemboli of cryptogenic origin compared to non-cardioembolic thrombemboli<br><br>Older thrombemboli (>1 day) contain more neutrophils than fresh thrombemboli (***p* = 0.0002**)	
Novotny et al 2020 [20]	Prospective<br><br>June 2005 – December 2013 (patients with AMI)<br><br>October 2010 – September	AIS	NE<br>H3cit<br>Extracellular DNA	AIS patients with anterior circulation stroke undergone MT<br><br><u>Group 1:</u><br>Patients with AIS<br>n = 71<br>mean age = 69.9 ± 13.8	NIHSS<br><br>mRS<br><br>TICI Score<br><br>TOAST classification	Thrombi	During mechanical recanalization	Amounts of neutrophils (***p* = 0.56**) were similar between AIS and AMI thrombi.<br><br>NETs were present in 100% of patients with AIS and 20.8% of patients with AMI.	Only patients with successful thrombus aspiration were eligible for enrollment.

	2012 (AIS patients)			<u>Group 2:</u><br>Patients with AMI (Acute Myocardial Infarction)<br>*n* = 72<br>mean age = 61.5 ± 10.9				Thrombi classified as TOAST-1 presented fewer NETs (***p* = 0.0476**) and less netting neutrophils (***p* = 0.0184**) compared with TOAST-2 patients. In thrombi classified as TOAST-5, more NETs were present compared with TOAST-1 (***p* = 0.0485)**<br><br>NETs were associated with interventional markers representing thrombus stability and with worse clinical outcome based on the NIHSS post-assessment score (***p* = 0.0003**) and mRS until 90 days (***p* = 0.006**)	

Lim et al.<br>2020<br>[13]	Prospective<br><br>October 2017 –<br>August 2018	AIS	DNA-histone complex<br><br>dsDNA<br><br>Neutrophil	<u>Group 1:</u><br>Patients newly diagnosed with ACS (acute coronary syndrome)<br>*n* = 37<br>mean age = 67.1 ± 10.1<br><br><u>Group 2:</u><br>Patients newly diagnosed with AIS<br>*n* = 58<br>mean age = 68.2 ± 11.3<br><br><u>Group 3:</u><br>Healthy controls<br>*n* = 25<br>mean age = 66.4 ± 7.1	NIHSS	Residual blood samples without any additional blood collection	Not mentioned	Concentrations of dsDNA were statistically higher in patients with ACS or AIS than those in the control group ***p*< 0.001**<br><br>In the univariable and multivariable analyses, statistically significant risk factors were **troponin I (TnI) level** and **dsDNA concentration** in the ACS group (***p* = 0.046**<br>***p*= 0.015** respectively) and only **dsDNA concentration** in the AIS group (***p* = 0.002**)	Relatively small sample size<br><br>Sampling in different time points
Zhou et al.<br>2021<br>[35]	Prospective<br>April 2017 –<br>March 2019	AIS	MPO-DNA<br><br>NE-DNA<br><br>H3Cit-DNA<br><br>cfDNA	<u>Group 1:</u><br>AIS patients with acute ICA occlusion who had undergone endovascular thrombectomy<br>*n* = 55	NIHSS<br><br>mRs<br><br>ASPECTS	Arterial blood samples<br><br>Thrombi	Healthy controls: during DSA	NET-specific markers were elevated on the plasma from carotid artery relative to plasma from the aorta or controls<br>***p* < 0.01**	Not mentioned

				mean age = 65.35 ± 11.5<br><br>Group 2:<br>Healthy controls<br>*n* = 35<br>mean age = 61.44 ± 9.89					
Cai et al. 2019 [34]	Prospective<br><br>1 January 2014 – 10 August 2018	AIS	Neutrophil count<br><br>Neutrophil to lymphocyte ratio (NLR) in the peripheral blood<br><br>Infarct sizes within 24h after symptom onset were analyzed	Group 1: Patients<br>*n* = 225<br>mean age 64.87 ± 0.60<br><br>*Group 1a:* Patients who had infection<br>*n* = 58<br><br>*Group 1b:* Patients who had not infection<br>*n* = 167<br><br>Group 2:<br>Healthy controls *n* = 56<br>age and gender-matched with patients	NIHSS	Peripheral blood	Within 24h after symptom onset	Higher neutrophils and neutrophil to lymphocyte ratio (NLR) in AIS patients with or without infection compared to controls **($p \leq 0.001$)**<br><br>In AIS patients without infection, NLR, was positively correlated with higher NIHSS scores **($p = 0.0006$)**<br><br>In patients with infarction > 1.5cm, both neutrophil counts (**$p = 0.0208$**) and NLR (**$p = 0.0447$**) positively correlated with patients' infarct sizes	Not mentioned

Bang et al. 2019 [10]	Prospective cohort study<br><br>Not mentioned	AIS	Plasma DNA (CD62P)<br><br>Neutrophil ($\times 10^3$/mL)	<u>Group 1:</u> cancer-related stroke n = 38 mean age = 65.4 ±- 10.3<br><br><u>Group 2:</u> healthy controls n = 33 mean age = 56.3 ± 9.4<br><br><u>Group 3:</u> cancer controls n = 27 mean age = 57 ± 9.9<br><br><u>Group 4:</u> stroke controls n = 40 mean age = 72.6 ± 10.4	Not mentioned	Blood sample	7 days after acute stroke due to embolic stroke of unknown sources	In multiple regression analyses, increased plasma DNA levels were associated with cancer-related stroke (odds ratio = 11.65 for the highest quartile; 95% CI, 3.199–42.46) and D-dimer levels of ≥2 μg/mL (odds ratio = 19.09 for highest quartile; 95% CI, 4.143–87.95) after adjusting for possible confounders.	Small sample size<br><br>Need for long follow-up study among cancer-related stroke patients<br><br>Association of NETs with cancer-unrelated thrombosis
Farkas et al. 2019 [18]	Prospective<br><br>2014–2016	AIS	Extracellular DNA<br><br>H3cit	<u>Group 1:</u> AIS patients n = 78<br><br><u>Group 2:</u>	Not mentioned	Thrombi	During acute therapeutic catheter interventions in CAD, open	Extracellular DNA content of AIS thrombi is similar to CAD and 2.5-fold lower than in PAD thrombi, ***p* = 0.013**	Heterogeneity of thrombi, especially if we consider the old age of some peripheral clots

				CAD patients<br>*n* = 66<br><br><u>Group 3:</u><br>PAD patients *n* = 64<br><br>Age not mentioned			surgery in PAD, or via stent-retriever thrombectomy	No difference was observed between H3cit content at different vascular locations.<br><br>The NET content of thrombi correlated parabolically with systemic inflammatory markers and positively with patients' age.	Numerous factors may affect thrombus structure, and thus it is hard to evaluate the effects of only one isolated determinant
Arroyo et al. 2018 [37]	Prospective<br><br>Not mentioned	AIS	cfDNA<br><br>NE plasma levels	<u>Group 1:</u><br>Steadily anticoagulated atrial fibrillation patients<br>*n* = 336<br>median age = 69<br><br><u>Group 2:</u><br>Healthy controls<br>*n* = 100<br>median age = 54 years	CHA2DS2-VASc score<br><br>CHA2DS2-VASc+NE	Blood samples	upon admission	cfDNA of 54.4±1.95 ng/mL. was significantly higher in the plasma of AF patients compared to controls **($p < 0.001$)**<br><br>NE level >55.29 ng/mL was associated with a higher risk of all-cause mortality (**$p < 0.001$**), cardiovascular mortality (**$p = 0.020$**), composite cardiovascular events (**$p = 0.005$**), and ischemic stroke (**$p = 0.009$**)	Single center, population<br><br>All AF patients had good anticoagulation control => results should only be extrapolated to clinically similar patients

Ducroux et al. 2018 [16]	Prospective<br><br>December 2015 – December 2016	AIS	Extracellular DNA<br><br>MPO<br><br>NE activity<br><br>H3Cit	AIS patients treated with endovascular therapy<br>n = 108<br>mean age = 69 ± 15.83	NIHSS score<br><br>TICI score<br><br>TOAST classification	Thrombi	At the end of MT	NETs were found in all 34 thrombi analyzed in histology.<br><br>There was no significant correlation between NETs content and stroke pathogenesis, 3-month functional outcome, or final Thrombolysis in Cerebral Infarction (TICI) score<br><br>NETs content is associated with endovascular procedure length and device number of passes.<br><br>tPA and DNAse 1 coadministration accelerates ex vivo thrombolysis compared with tPA or DNAse 1 alone.	Not mentioned

Valles et al. 2017 [3]	Prospective<br><br>Not Mentioned	AIS	cfDNA<br><br>citH3	<u>Group 1:</u><br>AIS patients<br>*n* = 243 (follow up one year after hospital discharge 179)<br>mean age = 70.7 ± 12.2<br><u>Group 2:</u><br>Healthy controls<br>*n* = 27	NIHSS<br><br>mRS	Peripheral blood	Patients: within the first 72 h after the onset of the acute event<br><br>Healthy controls: after a night fast	levels of **H3cit** in AIS patients (0.080 ± 0.002 [0.040–0.284] ***p* < 0.0001**, **cfDNA** (432.11 ± 9.95 [145.6–1227.5], ***p* < 0.0001**, and **nucleosomes** (0.329 ± 0.015 [0.07–1.87] ***p* = 0.002** were significantly higher than in controls Positive correlation between age and citH3 only. cfDNA levels were slightly higher in women (***p* = 0.047**).<br><br>All three markers of NETs were significantly elevated in patients with a history of AF<br><br>Positive correlation between NIHSS score at onset and the levels of all three NET markers. When patients were stratified according to NIHSS score at onset (<14 or ≥14), those with higher scores had	Not Mentioned

								significantly higher levels of all three NETs [citH3 (p = 0.000), cfDNA (***p* = 0.017**), and nucleosomes (***p* = 0.05**)<br><br>At discharge, the levels of all three NET markers were positively correlated with the NIHSS score.<br><br>Levels of cfDNA and CitH3 were higher in the patients with cardioembolic stroke.<br><br>Patients who died at one-year clinical follow-up from all causes had significantly higher citH3 at onset than survivors	
Laridan et al.2017 [40]	Prospective<br><br>Not mentioned	AIS	H3cit<br>NE	Group 1:<br>Ischemic stroke patients after thrombectomy procedure	NIHSS at admission and discharge<br>TOAST criteria	Thrombi	During thrombectomy procedure	abundant presence of NETs in ischemic stroke thrombi<br><br>Older thrombi (>1 day) contained significantly	Only thrombi that did not dissolve spontaneously or after t-PA administration and that could be

				n = 68<br>mean age = 69.1 ± 18.3<br><br>*Group 1a:* fresh thrombi (<1 day)<br>*n* = 32<br><br>*Group 1b:* older thrombi (>1 day)<br>*n* = 36				higher amounts of neutrophils compared to fresh thrombi (<1 day) (**$p < 0.001$)**<br><br>Thrombi of cardioembolic origin contained nearly double the number of NETs (3.07% ± 2.21%) compared to non-cardioembolic thrombi (1.57% ± 1.23%) (**$p < 0.05$)**<br><br>Older thrombi showed significantly higher amounts of H3Cit compared to fresh thrombi (3.23% ± 2.76% versus 2.03% ± 1.63%) (**$p < 0.05$**)<br><br>DNase 1 can promote ex vivo stroke thrombus dissolution **($p < 0.01$)**	successfully retrieved via thrombectomy were available for study<br><br>Low number of patients with non-cardioembolic stroke does not allow for robust conclusions regarding stroke etiology

Thålin et al. 2016 [17]	Prospective, observational case-control study<br><br>April 2012 – December 2014	AIS	cfDNA<br><br>MPO<br>H3Cit	Group 1:<br>Patients with ischemic stroke and hsTnT > 40 ng/L (ref value < 15 ng/L)<br>*n* = 12<br><br>Group 2:<br>Patients with ischemic stroke and hsTnT ≤ 15 ng/L as **control patients** *n* = 19<br><br>Group 3:<br>Healthy volunteers matched on sex and age within a five-year interval, were recruited as references for plasma analyses.<br>*n* = 10<br><br>Group 4:<br>Patients with elevated hsTnT and malignancies	NIHSS	Blood samples<br><br>Autopsy and histopathology (Group 4 patients)	Within two days of admission	A procoagulant state and an increase of the NET-specific marker citrullinated histone H3 (H3Cit) were found in the plasma of patients with elevated hsTnT compared to patients with normal levels.***p* < 0.001**<br><br>Plasma analyses in cancer patients showed even higher H3Cit levels (***p* < 0.001**), and an increase in granulocyte colony-stimulating factor, known to prime neutrophils towards NETosis.	Small sample size

				revealed widespread arterial H3Cit-positive Microthrombosis<br>*n* = 3					
Kitano et al. 2021 [39]	Multicenter retrospective study<br><br>January 2015 – December 2019	AIS	Citrullinated histone H3	AIS patients undergone MT, *n* = 54<br><u>Group 1:</u> Fresh thrombus (<1 day)<br>*n* = 43 (23%)<br>mean age =77 (67–84)<br><br><u>Group 2:</u><br>Older thrombi (>1 day) including lytic thrombus (1–5 days)<br>*n* = 131 (71%) and organized thrombus (>5 days)<br>*n* = 11 (6%)<br>*n* = 142<br>mean age = 80 (70–84)	mRs<br><br>NIHSS<br><br>ASPECTS<br><br>eTICI	Thrombi	During mechanical thrombectomy	Older thrombi had greater extent of NETosis (***p* = 0.006**).<br><br>More more device passes before reperfusion were necessary for older thrombi (***p* < 0.001**) and were associated with poorer functional outcomes (adjusted common odds ratio: 0.49; 95% CI: 0.24–0.99).	Patients with thrombi unavailable for evaluation were excluded.<br><br>The exact timing of thrombus content ormation (primary or possibly secondary thrombi)was not clear.
Puy et al 2021 [25]	Post-Mortem Study 2005–2019	sICH	Myeloperoxidase<br><br>Histone H3	sICH patients who came to autopsy within the first month after stroke	Not mentioned	Autopsy (brains)	Within 12 to 36 hours after death	Neutrophils were increased in all cases (*n* = 14/14) compared to control	Elderly patients and sICH were associated with underlying cerebral

				onset<br>$n = 14$<br>median age = 78 (76–85)<br><br>From stroke onset to time of death:<br><br>Group 1:<br>72 hours ($n = 2$)<br><br>Group 2:<br>4–7 days ($n = 4$)<br><br>Group 3:<br>8–15 days ($n = 5$)<br><br>Group 4:<br>>15 days<br>($n = 3$)				contralateral sections in which no neutrophils were observed within the tissue, **$p < 0.0001$**<br><br>NETs were found in 7/14 cases.<br><br>Both neutrophils and NETs were detected within the hematoma but also, in the surrounding tissue.<br><br>The appearance of neutrophils and NETs was time-dependent, following a two-wave pattern: during the first 72 hours and between 8–15 days after ICH onset.(**$p = 0.016$**)	amyloid angiopathy (CAA) in 13/14 cases<br><br>Did not perform an exhaustive examination or quantification of the entire sICH lesion

Abbreviations:.

ACS: Acute Coronary Syndrome, **AIS:** Acute ischemic stroke, **AF:** atrial fibrillation, **aSAH:** aneurysmal subarachnoid hemorrhage ,**CAD:** Coronary Artery Disease, **CLA**: Clot Lysis Assay, **cfDNA:**cell-free DNA **DCI:** Delayed cerebral ischemia, **EVT:** endovascular thrombectomy, **GCS:** Glasgow Coma Scale, **H3cit:** Citrullinated histone H3**, ICH:** intracerebral hemorrhage**, IQR:** interquartile ranges, **IS:** ischemic stroke, **iTTP:** immune Thrombotic Thrombopenic Porphyra, **IVT:** intravenous thrombolysis**, LDG:** Low-density granulocytes**, LVO:** large vessel occlusion, ,**MPO:** myeloperoxidase, **mRS:** modified Rankin Scale, **MT:** Mechanical Thrombectomy, **NE:** neutrophil elastase, **NETs:** neutrophil extracellular traps, **NIHSS:** National Institutes of Health Stroke Scale, **NLR:** neutrophil-to-lymphocyte ratio**, PAD:** Peripheral Artery Disease, **SAH:** subarachnoid hemorrhage**, sICH:** symptomatic intracerebral hemorrhage**, TIA:** transient ischemic attack, **TICI**: Thrombolysis in cerebral infarction, **TOAST:** Trial of Org 10172 in Acute Stroke Treatment.
